# Estimated Effectiveness of COVID-19 Vaccines Against Omicron or Delta Symptomatic Infection and Severe Outcomes

**DOI:** 10.1001/jamanetworkopen.2022.32760

**Published:** 2022-09-22

**Authors:** Sarah A. Buchan, Hannah Chung, Kevin A. Brown, Peter C. Austin, Deshayne B. Fell, Jonathan B. Gubbay, Sharifa Nasreen, Kevin L. Schwartz, Maria E. Sundaram, Mina Tadrous, Kumanan Wilson, Sarah E. Wilson, Jeffrey C. Kwong

**Affiliations:** 1 Public Health Ontario, Toronto, Ontario, Canada; 2 ICES, Toronto, Ontario, Canada; 3 Dalla Lana School of Public Health, University of Toronto, Toronto, Ontario, Canada; 4 Centre for Vaccine Preventable Diseases, University of Toronto, Toronto, Ontario, Canada; 5 Institute of Health Policy, Management and Evaluation, University of Toronto, Toronto, Ontario, Canada; 6 School of Epidemiology and Public Health, University of Ottawa, Ottawa, Ontario, Canada; 7 Children’s Hospital of Eastern Ontario Research Institute, Ottawa, Ontario, Canada; 8 Department of Laboratory Medicine and Pathobiology, University of Toronto, Toronto, Ontario, Canada; 9 Center for Clinical Epidemiology and Population Health, Marshfield Clinic Research Institute, Marshfield, Wisconsin; 10 Women’s College Hospital, Toronto, Ontario, Canada; 11 Leslie Dan Faculty of Pharmacy, University of Toronto, Toronto, Ontario, Canada; 12 Department of Medicine, University of Ottawa, Ottawa, Ontario, Canada; 13 Bruyère Research Institute, Ottawa, Ontario, Canada; 14 Ottawa Hospital Research Institute, Ottawa, Ontario, Canada; 15 Department of Family and Community Medicine, University of Toronto, Toronto, Ontario, Canada; 16 University Health Network, Toronto, Ontario, Canada

## Abstract

**Importance:**

The incidence of SARS-CoV-2 infection, including among individuals who have received 2 doses of COVID-19 vaccine, increased substantially following the emergence of the Omicron variant in Ontario, Canada. Understanding the estimated effectiveness of 2 or 3 doses of COVID-19 vaccine against outcomes associated with Omicron and Delta infections may aid decision-making at the individual and population levels.

**Objective:**

To estimate vaccine effectiveness (VE) against symptomatic infections due to the Omicron and Delta variants and severe outcomes (hospitalization or death) associated with these infections.

**Design, Setting, and Participants:**

This test-negative case-control study used linked provincial databases for SARS-CoV-2 laboratory testing, reportable disease, COVID-19 vaccination, and health administration in Ontario, Canada. Participants were individuals aged 18 years or older who had COVID-19 symptoms or severe outcomes (hospitalization or death) and were tested for SARS-CoV-2 between December 6 and 26, 2021.

**Exposures:**

Receipt of 2 or 3 doses of the COVID-19 vaccine and time since last dose.

**Main Outcomes and Measures:**

The main outcomes were symptomatic Omicron or Delta infection and severe outcomes (hospitalization or death) associated with infection. Multivariable logistic regression was used to estimate the effectiveness of 2 or 3 COVID-19 vaccine doses by time since the latest dose compared with no vaccination. Estimated VE was calculated using the formula VE = (1 – [adjusted odds ratio]) × 100%.

**Results:**

Of 134 435 total participants, 16 087 were Omicron-positive cases (mean [SD] age, 36.0 [14.1] years; 8249 [51.3%] female), 4261 were Delta-positive cases (mean [SD] age, 44.2 [16.8] years; 2199 [51.6%] female), and 114 087 were test-negative controls (mean [SD] age, 42.0 [16.5] years; 67 884 [59.5%] female). Estimated VE against symptomatic Delta infection decreased from 89% (95% CI, 86%-92%) 7 to 59 days after a second dose to 80% (95% CI, 74%-84%) after 240 or more days but increased to 97% (95% CI, 96%-98%) 7 or more days after a third dose. Estimated VE against symptomatic Omicron infection was 36% (95% CI, 24%-45%) 7 to 59 days after a second dose and 1% (95% CI, –8% to 10%) after 180 days or longer, but 7 or more days after a third dose, it increased to 61% (95% CI, 56%-65%). Estimated VE against severe outcomes was high 7 or more days after a third dose for both Delta (99%; 95% CI, 98%-99%) and Omicron (95%; 95% CI, 87%-98%).

**Conclusions and Relevance:**

In this study, in contrast to high estimated VE against symptomatic Delta infection and severe outcomes after 2 doses of COVID-19 vaccine, estimated VE was modest and short term against symptomatic Omicron infection but better maintained against severe outcomes. A third dose was associated with improved estimated VE against symptomatic infection and with high estimated VE against severe outcomes for both variants. Preventing infection due to Omicron and potential future variants may require tools beyond the currently available vaccines.

## Key Points

**Question:**

What is the estimated effectiveness of COVID-19 vaccines for preventing symptomatic infections due to the Omicron and Delta variants and severe outcomes (hospitalization or death) associated with these infections?

**Findings:**

In this test-negative case-control study of 134 435 adults in Ontario, Canada, the estimated effectiveness of 2 doses of COVID-19 vaccine was high against symptomatic Delta infection and severe outcomes and was lower against symptomatic Omicron infection. After a third dose, estimated vaccine effectiveness against Omicron was 61% for symptomatic infection and 95% for severe outcomes.

**Meaning:**

The findings suggest that 3 doses of COVID-19 vaccine may protect against symptomatic Omicron infection and severe outcomes, but other measures are also likely needed to prevent Omicron infection.

## Teaser

This case-control study estimates the effectiveness of COVID-19 vaccines against symptomatic infections due to the Delta and Omicron variants and severe outcomes associated with these infections among adults in Ontario, Canada.

## Introduction

The World Health Organization declared Omicron a COVID-19 variant of concern on November 26, 2021, owing to its highly transmissible nature and risk of immune evasion.^1^ In Ontario, Canada, the first detected case of Omicron was identified on November 22, 2021; within weeks, Omicron accounted for most new cases. Despite high 2-dose COVID-19 vaccine coverage in Ontario (88% among individuals aged ≥18 years by mid-December 2021),^2^ the rate of cases among fully vaccinated individuals increased substantially during this period.^3^

A reduction in neutralizing antibodies against Omicron following second and third doses of mRNA vaccines has been established,^4,5,6,7^ but clinical data evaluating vaccine performance are still emerging.^8,9,10,11,12,13,14,15,16^ The objective of this study was to estimate vaccine effectiveness (VE) against symptomatic infection due to the Omicron or Delta variants and severe outcomes (hospitalization or death) associated with these infections in Ontario.

## Methods

### Study Population, Setting, Design, and Data Sources

In this case-control study, we used a test-negative study design and linked provincial databases in Ontario, Canada, to estimate VE. We linked provincial SARS-CoV-2 laboratory testing, reportable disease, COVID-19 vaccination, and health administrative databases using unique encoded identifiers and analyzed them at ICES, a not-for-profit provincial research institute.^17^ ICES is a prescribed entity under Ontario’s Personal Health Information Protection Act, section 45 of which authorizes ICES to collect personal health information without consent for analysis or compiling statistical information with respect to the management of, evaluation or monitoring of, allocation of resources to, or planning for all or part of the health system. Projects that use data collected by ICES under section 45 of the Personal Health Information Protection Act and use no other data are exempt from research ethics board review. The use of the data in this study was authorized under section 45 and was approved by the ICES Privacy and Legal Office. We followed the Strengthening the Reporting of Observational Studies in Epidemiology (STROBE) reporting guideline for case-control studies.^18^

We included individuals in Ontario who had COVID-19 symptoms, were aged 18 years or older, had provincial health insurance, and had a SARS-CoV-2 real-time reverse transcription–polymerase chain reaction (PCR) test between December 6 and 26, 2021. Our study period aligned with a provincial initiative to universally screen positive specimens to differentiate Omicron and Delta cases and was before restrictions in laboratory-based PCR test eligibility announced on December 30, 2021.^19^ We excluded long-term care residents, individuals who had received only 1 dose or 4 doses of COVID-19 vaccine or who had received a second dose less than 7 days before being tested, individuals who tested positive for SARS-CoV-2 within the previous 90 days, individuals who had received 2 or 3 doses of ChAdOx1 vaccine (AstraZeneca) because estimated VE for that primary schedule is known to be lower and the product has rarely been used as a third dose,^20^ individuals who had received a vaccine not authorized by Health Canada for any dose, and individuals who had received the Ad.26.COV2.S vaccine (Janssen/Johnson & Johnson; although approved for use, <0.1% of the Ontario population received this vaccine^21^) (eFigure in the Supplement).

### Outcomes

We identified individuals with confirmed SARS-CoV-2 infection using reportable disease and laboratory data. To estimate VE against symptomatic infection, we restricted our analysis to individuals who had at least 1 COVID-19–related symptom (self-reported or measured) at the time of testing, as identified in the laboratory testing data.^22^ The specimen collection date was used as the index date. We used reportable disease data to identify severe outcomes, defined as hospital admission or death. Public health units were directed to record hospitalizations and deaths associated with COVID-19 in the reportable disease system. For symptomatic individuals who tested negative for SARS-CoV-2 repeatedly during the study period and were considered controls (for both outcomes), we randomly selected 1 test with a negative result.

Specimens positive for SARS-CoV-2 identified through whole genome sequencing as B.1.1.529 lineage or found to have S-gene target failure (SGTF; a proxy measure for Omicron, resulting from the amino acid 69-70 spike deletion that does not occur with Delta) were considered to be infected with Omicron. Specimens sequenced as B.1.617 lineage or found to be negative for SGTF were considered to be infected with Delta. Individuals with unknown or inconclusive SGTF results were excluded.^23^ Between December 6 and 24, 2021, all specimens with a positive PCR result (and a cycle threshold value ≤35) should have been sent for testing using ThermoFisher TaqPath COVID-19 PCR to identify SGTF; as such, we selected our primary study period to approximate the period of universal screening for SGTF. Before December 20, 2021, SGTF-positive specimens with cycle threshold values of 30 or less also underwent whole genome sequencing. In Ontario, the estimated sensitivity of SGTF compared with whole genome sequencing for detecting Omicron among samples with a cycle threshold value of 30 or less was 98.9%, and the specificity was 99.9%.^23^

### COVID-19 Vaccination

To date, Ontario has primarily used 3 products (BNT162b2 [Pfizer-BioNTech], mRNA-1273 [Moderna], and ChAdOx1) in its COVID-19 vaccination program. Owing to fluctuating vaccine supply, varying dosing intervals and mixed vaccine schedules were used. We classified individuals based on the number of doses received and the timing of these doses relative to the index date. We included individuals who received at least 1 mRNA vaccine for the primary 2-dose series (because a mixed schedule consisting of ChAdOx1 and an mRNA vaccine was demonstrated to have similar estimated VE as 2 mRNA vaccines).^20^ For the third dose, we included individuals who received any mRNA vaccine and also stratified by the specific third-dose product. All comparisons included individuals who had not yet received any doses (ie, unvaccinated) by the index date as the reference group.

Third-dose eligibility in Ontario began in August 2021 and expanded gradually.^24^ Initially, only moderately or severely immunocompromised individuals were eligible to receive a third dose as part of an extended primary series. Third doses (ie, boosters) were then provided to residents of higher-risk, congregate settings for older adults (eg, long-term care homes, high-risk retirement homes). In early October 2021, older adults living in other congregate care settings, including all remaining retirement homes, became eligible. All individuals aged 70 years or older and health care workers became eligible on November 6, 2021; individuals aged 50 years or older, on December 13, 2021; and those aged 18 years or older, on December 18, 2021.^25^ The standard interval for third-dose eligibility was generally 168 or more days following a second dose but was shortened to 84 or more days on December 15, 2021.^26^

### Covariates

We obtained information on each individual’s age (in 10-year age bands); sex; public health unit region of residence; number of SARS-CoV-2 PCR tests during the 3 months before December 14, 2020 (as a proxy for health care worker status based on the start date of the provincial COVID-19 vaccine program); SARS-CoV-2 infection more than 90 days before the index date (to account for protection conferred from past infection)^27^; comorbidities associated with increased risk of severe COVID-19; influenza vaccination status during the 2019-2020 and/or 2020-2021 influenza seasons (as a proxy for health behaviors); and neighborhood-level information on median household income, proportion of the working population employed as nonhealth essential workers, mean number of persons per dwelling, and proportion of the population who self-identified as belonging to a visible minority group (non-Aboriginal peoples who are not White, consisting mainly of Arab, Black, Chinese, Filipino, Korean, Japanese, Latin American, South Asian, Southeast Asian, and West Asian groups).^28^ These data were included owing to potential confounding. These definitions and the databases used are described elsewhere.^22^

### Statistical Analysis

We calculated means (continuous variables) and frequencies (categorical variables) of baseline characteristics and compared test-positive cases (separately for Omicron and Delta) with test-negative controls using standardized differences. We used multivariable logistic regression to estimate adjusted odds ratios (aORs) comparing the odds of vaccination in each time-since-second-or-third-dose interval among cases with the odds among controls while adjusting for all aforementioned covariates and a categorical variable representing the week of the test. Estimated VE was calculated using the formula VE = (1 – aOR) × 100%. We did not report VE estimates when the width of the 95% CI was 100 percentage points or greater.

We performed a sensitivity analysis for severe outcomes using a less specific definition of Omicron infection to allow for a larger sample size. In this analysis, cases diagnosed from December 21, 2021, onward (when the prevalence of SGTF in the province was >90%) with no or inconclusive SGTF results were considered to be Omicron infection.

All analyses were conducted using SAS, version 9.4 (SAS Institute Inc). All tests were 2-sided and used *P* < .05 as the level of statistical significance.

## Results

Between December 6 and 26, 2021, the study population included 134 435 adults, of whom 16 087 were Omicron-positive cases (mean [SD] age, 36.0 [14.1] years; 8249 [51.3%] female; 7838 [48.7%] male), 4261 were Delta-positive cases (mean [SD] age, 44.2 [16.8] years; 2199 [51.6%] female; 2062 [48.4%] male), and 114 087 were test-negative controls (mean [SD] age, 42.0 [16.5] years; 67 884 [59.5%] female; 46 203 [40.5%] male). Compared with controls, Omicron cases were younger, more likely to be male, less likely to have any comorbidities, less likely to have received an influenza vaccine, less likely to have previously tested positive for SARS-CoV-2, and less likely to have received a third vaccine dose. Cases of Omicron infection were also more likely to have occurred during the last week of the study period compared with controls (Table 1).

**Table 1.** Characteristics of Omicron and Delta Cases and SARS-CoV-2–Negative Controls^a^

Characteristic	SARS-CoV-2–negative controls (n = 114 087)^b^	Omicron cases (n = 16 087)^b^	Standardized difference^c^^,^^d^	Delta cases (n = 4261)^b^	Standardized difference^c^^,^^e^
Age, mean (SD), y	42.0 (16.5)	36.0 (14.1)	0.39	44.2 (16.8)	0.13
Age group, y					
18-29	30 947 (27.1)	6813 (42.4)	0.32	960 (22.5)	0.11
30-39	28 387 (24.9)	3467 (21.6)	0.08	941 (22.1)	0.07
40-49	19 007 (16.7)	2821 (17.5)	0.02	851 (20.0)	0.09
50-59	16 695 (14.6)	1922 (11.9)	0.08	679 (15.9)	0.04
60-69	11 109 (9.7)	723 (4.5)	0.21	450 (10.6)	0.03
70-79	5386 (4.7)	233 (1.4)	0.19	269 (6.3)	0.07
≥80	2556 (2.2)	108 (0.7)	0.13	111 (2.6)	0.02
Sex					
Female	67 884 (59.5)	8249 (51.3)	0.17	2199 (51.6)	0.16
Male	46 203 (40.5)	7838 (48.7)	0.17	2062 (48.4)	0.16
Any comorbidity^f^	49 201 (43.1)	5844 (36.3)	0.14	1898 (44.5)	0.03
SARS-CoV-2 tests within 3 mo before Dec 14, 2020, No.					
0	83 457 (73.2)	12 448 (77.4)	0.10	3473 (81.5)	0.20
1	21 860 (19.2)	2782 (17.3)	0.05	615 (14.4)	0.13
≥2	8770 (7.7)	857 (5.3)	0.10	173 (4.1)	0.15
Receipt of 2019-2020 and/or 2020-2021 influenza vaccine	40 597 (35.6)	4012 (24.9)	0.23	1053 (24.7)	0.24
Public health unit region^g^					
Central East	9139 (8.0)	831 (5.2)	0.11	373 (8.8)	0.03
Central West	21 191 (18.6)	3899 (24.2)	0.14	864 (20.3)	0.04
Durham	7690 (6.7)	1050 (6.5)	0.01	191 (4.5)	0.10
Eastern	7010 (6.1)	865 (5.4)	0.03	256 (6.0)	0.01
North	7518 (6.6)	265 (1.6)	0.25	313 (7.3)	0.03
Ottawa	5716 (5.0)	979 (6.1)	0.05	99 (2.3)	0.14
Peel	12 592 (11.0)	2547 (15.8)	0.14	526 (12.3)	0.04
Southwest	12 888 (11.3)	998 (6.2)	0.18	814 (19.1)	0.22
Toronto	21 732 (19.0)	3102 (19.3)	0.01	542 (12.7)	0.17
York	8148 (7.1)	1476 (9.2)	0.07	266 (6.2)	0.04
Household income quintile^g^^,^^h^					
1	17 605 (15.4)	1908 (11.9)	0.10	786 (18.4)	0.08
2	20 783 (18.2)	2532 (15.7)	0.07	784 (18.4)	0
3	22 516 (19.7)	3085 (19.2)	0.01	840 (19.7)	0
4	24 861 (21.8)	3773 (23.5)	0.04	906 (21.3)	0.01
5	27 795 (24.4)	4707 (29.3)	0.11	925 (21.7)	0.06
Essential workers quintile^g^^,^^i^					
1	26 896 (23.6)	4539 (28.2)	0.11	648 (15.2)	0.21
2	28 043 (24.6)	4487 (27.9)	0.08	973 (22.8)	0.04
3	22 737 (19.9)	3057 (19.0)	0.02	903 (21.2)	0.03
4	19 614 (17.2)	2272 (14.1)	0.08	834 (19.6)	0.06
5	16 034 (14.1)	1621 (10.1)	0.12	866 (20.3)	0.17
Persons per dwelling quintile^g^^,^^j^					
1	21 183 (18.6)	2523 (15.7)	0.08	711 (16.7)	0.05
2	19 168 (16.8)	1934 (12.0)	0.14	738 (17.3)	0.01
3	15 197 (13.3)	1919 (11.9)	0.04	577 (13.5)	0.01
4	27 853 (24.4)	4143 (25.8)	0.03	1026 (24.1)	0.01
5	29 879 (26.2)	5443 (33.8)	0.17	1169 (27.4)	0.03
Self-identified visible minority group quintile^g^^,^^k^^,^^l^					
1	16 930 (14.8)	1439 (8.9)	0.18	779 (18.3)	0.09
2	20 294 (17.8)	2235 (13.9)	0.11	882 (20.7)	0.07
3	22 577 (19.8)	3408 (21.2)	0.03	841 (19.7)	0
4	26 063 (22.8)	4250 (26.4)	0.08	795 (18.7)	0.10
5	27 463 (24.1)	4644 (28.9)	0.11	928 (21.8)	0.05
Week of test					
Dec 6 to Dec 12, 2021	31 103 (27.3)	729 (4.5)	0.65	1444 (33.9)	0.14
Dec 13 to Dec 19, 2021	41 090 (36.0)	5049 (31.4)	0.10	1830 (42.9)	0.14
Dec 20 to Dec 26, 2021	41 894 (36.7)	10 309 (64.1)	0.57	987 (23.2)	0.30
Prior positive SARS-CoV-2 test result	4257 (3.7)	117 (0.7)	0.20	10 (0.2)	0.25
Vaccination status					
Unvaccinated	4681 (4.1)	790 (4.9)	0.04	1251 (29.4)	0.72
Received 2-dose primary series only with ≥1 mRNA vaccine	91 305 (80.0)	13 813 (85.9)	0.16	2845 (66.8)	0.30
Received BNT162b2 for third dose	14 782 (13.0)	1225 (7.6)	0.18	134 (3.1)	0.37
Received mRNA-1273 for third dose	3319 (2.9)	259 (1.6)	0.09	31 (0.7)	0.16
Time since second dose, d					
7-59	2254 (2.0)	231 (1.4)	0.04	61 (1.4)	0.04
60-119	6769 (5.9)	1003 (6.2)	0.01	185 (4.3)	0.07
120-179	60 722 (53.2)	8543 (53.1)	0	1855 (43.5)	0.19
180-239	19 841 (17.4)	3817 (23.7)	0.16	668 (15.7)	0.05
≥240	1719 (1.5)	219 (1.4)	0.01	76 (1.8)	0.02
Time since third dose, d					
0-6	5963 (5.2)	804 (5.0)	0.01	74 (1.7)	0.19
7-59	11 283 (9.9)	638 (4.0)	0.23	74 (1.7)	0.35
≥60	855 (0.7)	42 (0.3)	0.07	17 (0.4)	0.05
Interval between first and second doses, d					
15-34	15 474 (13.6)	2273 (14.1)	0.02	425 (10.0)	0.11
35-55	37 972 (33.3)	6154 (38.3)	0.10	988 (23.2)	0.23
≥56	55 960 (49.1)	6870 (42.7)	0.13	1597 (37.5)	0.24
Interval between second and third doses, d					
≤111	232 (0.2)	18 (0.1)	0.02	8 (0.2)	0
112-167	1617 (1.4)	122 (0.8)	0.06	26 (0.6)	0.08
≥168	16 252 (14.2)	1344 (8.4)	0.19	131 (3.1)	0.41

^a^ Participants included adults with COVID-19–relevant symptoms who were tested for SARS-CoV-2 from December 6 to 26, 2021.

^b^ Data are presented as number (percentage) of participants unless otherwise indicated.

^c^ Standardized differences greater than 0.10 were considered clinically relevant.

^d^ Comparison of Omicron-positive cases with SARS-CoV-2–negative controls.

^e^ Comparison of Delta-positive cases with SARS-CoV-2–negative controls.

^f^ Comorbidities included chronic respiratory diseases, chronic heart diseases, hypertension, diabetes, immunocompromising conditions owing to underlying diseases or therapy, autoimmune diseases, chronic kidney disease, advanced liver disease, dementia/frailty, and history of stroke or transient ischemic attack.

^g^ The sum of counts does not equal the column total because of individuals with missing information (<1.0%) for this characteristic.

^h^ Household income quintiles have variable cutoff values in each city or census area to account for cost of living. A dissemination area being in quintile 1 means it is among the lowest 20% of dissemination areas in its city by income.

^i^ Percentage of people in the area working in the following occupations: sales and service occupations; trades, transport and equipment operators, and related occupations; natural resources, agriculture, and related production occupations; and occupations in manufacturing and utilities. Census counts for people were randomly rounded up or down to the nearest number divisible by 5, which caused some minor imprecision. Quintile 1 was 0% to <32.5%; quintile 2, 32.5% to <42.3%; quintile 3, 42.3% to <50.0%; quintile 4, 50.0% to <57.5%; and quintile 5, 57.5% to 100%.

^j^ Quintile 1 was 0 to 2.1; quintile 2, 2.2 to 2.4; quintile 3, 2.5 to 2.6; quintile 4, 2.7 to 3.0; and quintile 5, 3.1 to 5.7.

^k^ Census counts were randomly rounded up or down to the nearest number divisible by 5, which caused some minor imprecision. Quintile 1 was 0.0% to 2.23%; quintile 2, 2.24% to 7.53%; quintile 3, 7.54% to 18.68%; quintile 4, 18.69% to 43.52%; and quintile 5, 43.53% to 100%.

^l^ Non-Aboriginal peoples who are not White, consisting mainly of Arab, Black, Chinese, Filipino, Korean, Japanese, Latin American, South Asian, Southeast Asian, and West Asian groups.^28^

In contrast, Delta cases had some characteristics more similar to those of controls than to those of Omicron cases (eg, age, presence of comorbidities) and other characteristics more different from those of controls than those of Omicron cases, such as being more likely to be unvaccinated (Delta cases: 1251 [29.4%]; controls: 4681 [4.1%]; Omicron cases: 790 [4.9%]).

In the Omicron VE analyses, vaccinated individuals (ie, 2 or 3 doses) were more likely to be older, female, influenza vaccine recipients, and residents of neighborhoods with higher income and fewer essential workers than were unvaccinated individuals (Table 2). Recipients of a third dose were more likely to have comorbidities and to have been tested in the last week of the study period. We observed similar patterns for individuals in the Delta VE analyses (eTable 1 in the Supplement).

**Table 2.** Characteristics of Vaccinated and Unvaccinated Omicron Cases and SARS-CoV-2–Negative Controls^a^

Characteristic	Unvaccinated (n = 5471)^b^	2 Doses (n = 105 118)^b^	Standardized difference^c^^,^^d^	3 Doses (n = 19 585)^b^	Standardized difference^c^^,^^e^
Age, mean (SD), y	37.1 (15.1)	39.5 (15.1)	0.16	52.1 (18.8)	0.88
Age group, y					
18-29	2085 (38.1)	32 809 (31.2)	0.15	2866 (14.6)	0.55
30-39	1545 (28.2)	27 034 (25.7)	0.06	3275 (16.7)	0.28
40-49	796 (14.5)	18 474 (17.6)	0.08	2558 (13.1)	0.04
50-59	519 (9.5)	14 515 (13.8)	0.14	3583 (18.3)	0.26
60-69	298 (5.4)	8437 (8.0)	0.10	3097 (15.8)	0.34
70-79	137 (2.5)	2698 (2.6)	0	2784 (14.2)	0.43
≥80	91 (1.7)	1151 (1.1)	0.05	1422 (7.3)	0.27
Sex					
Female	2880 (52.6)	60 666 (57.7)	0.10	12 587 (64.3)	0.24
Male	2591 (47.4)	44 452 (42.3)	0.10	6998 (35.7)	0.24
Any comorbidity^f^	2088 (38.2)	41 929 (39.9)	0.04	11 028 (56.3)	0.37
SARS-CoV-2 tests within 3 mo before Dec 14, 2020, No.					
0	4435 (81.1)	78 274 (74.5)	0.16	13 196 (67.4)	0.32
1	780 (14.3)	19 987 (19.0)	0.13	3875 (19.8)	0.15
≥2	256 (4.7)	6857 (6.5)	0.08	2514 (12.8)	0.29
Receipt of 2019-2020 and/or 2020-2021 influenza vaccination	409 (7.5)	33 148 (31.5)	0.64	11 052 (56.4)	1.23
Public health unit region^g^					
Central East	531 (9.7)	7971 (7.6)	0.08	1468 (7.5)	0.08
Central West	1032 (18.9)	20 604 (19.6)	0.02	3454 (17.6)	0.03
Durham	309 (5.6)	7351 (7.0)	0.06	1080 (5.5)	0.01
Eastern	293 (5.4)	6268 (6.0)	0.03	1314 (6.7)	0.06
North	472 (8.6)	5802 (5.5)	0.12	1509 (7.7)	0.03
Ottawa	165 (3.0)	5398 (5.1)	0.11	1132 (5.8)	0.14
Peel	666 (12.2)	12 730 (12.1)	0	1743 (8.9)	0.11
Southwest	790 (14.4)	10 649 (10.1)	0.13	2447 (12.5)	0.06
Toronto	893 (16.3)	19 896 (18.9)	0.07	4045 (20.7)	0.11
York	283 (5.2)	8018 (7.6)	0.10	1323 (6.8)	0.07
Household income quintile^g^^,^^h^					
1	1253 (22.9)	15 708 (14.9)	0.20	2552 (13.0)	0.26
2	1182 (21.6)	18 931 (18.0)	0.09	3202 (16.3)	0.13
3	1100 (20.1)	20 940 (19.9)	0	3561 (18.2)	0.05
4	969 (17.7)	23 240 (22.1)	0.11	4425 (22.6)	0.12
5	923 (16.9)	25 829 (24.6)	0.19	5750 (29.4)	0.30
Essential workers quintile^g^^,^^i^					
1	783 (14.3)	24 992 (23.8)	0.24	5660 (28.9)	0.36
2	1042 (19.0)	26 526 (25.2)	0.15	4962 (25.3)	0.15
3	1142 (20.9)	20 899 (19.9)	0.02	3753 (19.2)	0.04
4	1205 (22.0)	17 717 (16.9)	0.13	2964 (15.1)	0.18
5	1231 (22.5)	14 333 (13.6)	0.23	2091 (10.7)	0.32
Persons per dwelling quintile^g^^,^^j^					
1	1011 (18.5)	18 695 (17.8)	0.02	4000 (20.4)	0.05
2	1132 (20.7)	16 613 (15.8)	0.13	3357 (17.1)	0.09
3	749 (13.7)	13 690 (13.0)	0.02	2677 (13.7)	0
4	1302 (23.8)	25 913 (24.7)	0.02	4781 (24.4)	0.01
5	1202 (22.0)	29 507 (28.1)	0.14	4613 (23.6)	0.04
Self-identified visible minority group quintile^g^^,^^k^^,^^l^					
1	992 (18.1)	14 273 (13.6)	0.12	3104 (15.8)	0.06
2	1014 (18.5)	17 891 (17.0)	0.04	3624 (18.5)	0
3	988 (18.1)	20 750 (19.7)	0.04	4247 (21.7)	0.09
4	1135 (20.7)	24 669 (23.5)	0.07	4509 (23.0)	0.06
5	1274 (23.3)	26 886 (25.6)	0.05	3947 (20.2)	0.08
Week of test					
Dec 6 to Dec 12, 2021	1580 (28.9)	27 714 (26.4)	0.06	2538 (13.0)	0.40
Dec 13 to Dec 19, 2021	1841 (33.7)	38 780 (36.9)	0.07	5518 (28.2)	0.12
Dec 20 to Dec 26, 2021	2050 (37.5)	38 624 (36.7)	0.02	11 529 (58.9)	0.44
Prior positive SARS-CoV-2 test result	261 (4.8)	3658 (3.5)	0.06	455 (2.3)	0.13
Vaccination status					
Unvaccinated	5471 (100)	NA	NA	NA	NA
Received 2-dose primary series only with ≥1 mRNA vaccine	NA	105 118 (100)	NA	NA	NA
Received BNT162b2 for third dose	NA	NA	NA	16 007 (81.7)	NA
Received mRNA-1273 for third dose	NA	NA	NA	3578 (18.3)	NA
Time since second dose, d					
7-59	NA	2485 (2.4)	NA	NA	NA
60-119	NA	7772 (7.4)	NA	NA	NA
120-179	NA	69 265 (65.9)	NA	NA	NA
180-239	NA	23 658 (22.5)	NA	NA	NA
≥240	NA	1938 (1.8)	NA	NA	NA
Time since third dose, d					
0-6	NA	NA	NA	6767 (34.6)	NA
7-59	NA	NA	NA	11 921 (60.9)	NA
≥60	NA	NA	NA	897 (4.6)	NA
Interval between first and second doses, d					
15-34	NA	14 355 (13.7)	NA	3392 (17.3)	NA
35-55	NA	39 792 (37.9)	NA	4334 (22.1)	NA
≥56	NA	50 971 (48.5)	NA	11 859 (60.6)	NA
Interval between second and third doses, d					
≤111	NA	NA	NA	250 (1.3)	NA
112-167	NA	NA	NA	1739 (8.9)	NA
≥168	NA	NA	NA	17 596 (89.8)	NA

Abbreviation: NA, not applicable.

^a^ Participants included adults with COVID-19–relevant symptoms who were tested for SARS-CoV-2 from December 6 to 26, 2021.

^b^ Data are presented as number (percentage) of participants unless otherwise indicated.

^c^ Standardized differences greater than 0.10 were considered clinically relevant.

^d^ Comparison of participants who had received 2 doses with unvaccinated participants.

^e^ Comparison of participants who had received 3 doses with unvaccinated participants.

^f^ Comorbidities included chronic respiratory diseases, chronic heart diseases, hypertension, diabetes, immunocompromising conditions owing to underlying diseases or therapy, autoimmune diseases, chronic kidney disease, advanced liver disease, dementia/frailty, and history of stroke or transient ischemic attack.

^g^ The sum of counts does not equal the column total because of individuals with missing information (<1.0%) for this characteristic.

^h^ Household income quintiles have variable cutoff values in each city or census area to account for cost of living. A dissemination area being in quintile 1 means it is among the lowest 20% of dissemination areas in its city by income.

^i^ Percentage of people in the area working in the following occupations: sales and service occupations; trades, transport and equipment operators, and related occupations; natural resources, agriculture, and related production occupations; and occupations in manufacturing and utilities. Census counts for people were randomly rounded up or down to the nearest number divisible by 5, which caused some minor imprecision. Quintile 1 was 0% to <32.5%; quintile 2, 32.5% to <42.3%; quintile 3, 42.3% to <50.0%; quintile 4, 50.0% to <57.5%; and quintile 5, 57.5% to 100%.

^j^ Quintile 1 was 0 to 2.1; quintile 2, 2.2 to 2.4; quintile 3, 2.5 to 2.6; quintile 4, 2.7 to 3.0; and quintile 5, 3.1 to 5.7.

^k^ Census counts were randomly rounded up or down to the nearest number divisible by 5, which caused some minor imprecision. Quintile 1 was 0.0% to 2.23%; quintile 2, 2.24% to 7.53%; quintile 3, 7.54% to 18.68%; quintile 4, 18.69% to 43.52%; and quintile 5, 43.53% to 100%.

^l^ Non-Aboriginal peoples who are not White, consisting mainly of Arab, Black, Chinese, Filipino, Korean, Japanese, Latin American, South Asian, Southeast Asian, and West Asian groups.^28^

After 2 doses of COVID-19 vaccine (with at least 1 mRNA vaccine), estimated VE against symptomatic Delta infection decreased steadily over time from 89% (95% CI, 86%-92%) 7 to 59 days after a second dose to 80% (95% CI, 74%-84%) after 240 days or longer but increased to 97% (95% CI, 96%-98%) 7 or more days after a third dose (Table 3 and Figure, A). Estimated VE was lower against symptomatic Omicron infection compared with Delta infection for the entire period and waned more rapidly from 36% (95% CI, 24%-45%) 7 to 59 days after a second dose to 1% (95% CI, –8% to 10%) 180 to 239 days after a second dose. Estimated VE against symptomatic Omicron infection was 61% (95% CI, 56%-65%) 7 or more days after a third dose and was similar regardless of the mRNA product administered.

**Table 3.** Estimates of Vaccine Effectiveness Against Symptomatic Omicron and Delta Infection or Severe Outcomes Associated With These Infections From December 6 to 26, 2021, by Time Since Latest Dose^a^

Dose, vaccine product	Time since latest dose, d	No.		Estimated vaccine effectiveness against Omicron, % (95% CI)	Vaccinated Delta cases, No.	Estimated vaccine effectiveness against Delta, % (95% CI)
		Vaccinated SARS-CoV-2–negative controls	Vaccinated Omicron cases			
**Symptomatic infection**							
First 2 doses: ≥1 mRNA vaccine	7-59	2254	231	36 (24 to 45)	61	89 (86 to 92)
	60-119	6769	1003	12 (3 to 21)	185	89 (87 to 90)
	120-179	60 722	8543	15 (8 to 22)	1855	88 (87 to 89)
	180-239	19 841	3817	1 (−8 to 10)	668	85 (83 to 87)
	≥240	1719	219	2 (−17 to 17)	76	80 (74 to 84)
Third dose						
Any mRNA vaccine	0-6	5963	804	36 (29 to 43)	74	94 (93 to 95)
	≥7	12 138	680	61 (56 to 65)	91	97 (96 to 98)
BNT162b2	0-6	4509	625	34 (26 to 41)	60	94 (92 to 95)
	≥7	10 273	600	60 (55 to 65)	74	97 (96 to 98)
mRNA-1273	0-6	1454	179	43 (32 to 52)	14	95 (92 to 97)
	≥7	1865	80	65 (55 to 72)	17	97 (95 to 98)
**Severe outcomes** ^b^							
First 2 doses: ≥1 mRNA vaccine	7-59	2254	≤5^c^	NE^d^	≤5^c^	94 (84 to 98)
	60-119	6769	6	NE^d^	≤5^c^	98 (94 to 99)
	120-179	60 722	31	75 (51 to 87)	40	99 (98 to 99)
	180-239	19 841	18	82 (62 to 91)	32	97 (96 to 98)
	≥240	1719	≤5^c^	NE^d^	≤5^c^	95 (85 to 99)
Third dose						
Any mRNA vaccine	0-6	5963	≤5^c^	91 (71 to 97)	6	99 (97 to 99)
	≥7	12 138	11	95 (87 to 98)	16	99 (98 to 99)
BNT162b2	0-6	4509	≤5^c^	88 (62 to 96)	6	98 (96 to 99)
	≥7	10 273	8	95 (87 to 98)	15	99 (98 to 99)
mRNA-1273	0-6	1454	0	100^e^	0	100^e^
	≥7	1865	≤5^c^	93 (74 to 98)	≤5^c^	100 (98 to 100)

Abbreviation: NE, not estimated.

^a^ Estimated vaccine effectiveness was calculated as 1 minus the adjusted odds ratio, multiplied by 100%. The estimates were adjusted for age (in 10-year age bands); sex; public health unit region of residence; number of SARS-CoV-2 polymerase chain reaction tests during the 3 months before December 14, 2020; SARS-CoV-2 infection more than 90 days before the index date; comorbidities; influenza vaccination status during the 2019-2020 and/or 2020-2021 influenza seasons; and neighborhood-level information on median household income, proportion of the working population employed as nonhealth essential workers, mean number of persons per dwelling, and proportion of the population who self-identified as belonging to a visible minority group.

^b^ Hospitalization or death.

^c^ Counts of vaccinated test-positive cases for each interval were suppressed because they were small cell counts (≤5), which cannot be disclosed because of privacy and data obligations.

^d^ Vaccine effectiveness estimate was suppressed owing to imprecision in the estimate and 95% CIs (ie, ≥100 percentage points).

^e^ Vaccine effectiveness was estimated as 100% based on 0 vaccinated test-positive cases.

**Figure.**
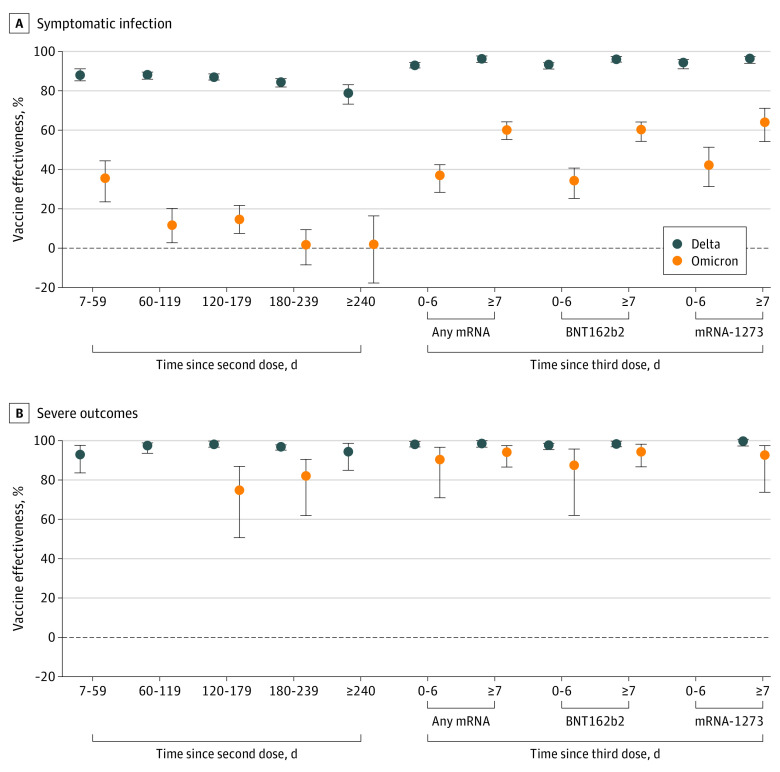
Estimates of Vaccine Effectiveness Against Symptomatic Omicron and Delta Infections and Severe Outcomes Associated With These Infections From December 6 to 26, 2021, by Time Since Latest Dose A and B, Markers indicate estimates, with vertical lines indicating 95% CIs. B, Severe outcomes included hospitalization and death. Omicron vaccine effectiveness estimates for 7 to 59 days, 60 to 119 days, and 240 days or more after the second dose are not presented owing to imprecision in the estimates and wide 95% CIs (ie, ≥100 percentage points). Vaccine effectiveness of the mRNA-1273 vaccine (Moderna) 0 to 6 days after the third dose was estimated as 100% based on 0 vaccinated test-positive hospitalized cases and therefore is not presented.

Estimated VE was higher against severe outcomes compared with symptomatic infection and did not have the same degree of waning (Figure, B). Estimated VE was also higher against severe outcomes associated with Delta than with Omicron in the time following a second dose, but 95% CIs were wide for some Omicron estimates. Estimated VE against severe outcomes 7 or more days following a third dose was slightly higher for Delta (99%; 95% CI, 98%-99%) than for Omicron (95%; 95% CI, 87%-98%). Model estimates for VE against Omicron or Delta symptomatic infection or severe outcomes are provided in eTables 2 and 3 in the Supplement.

When using a less specific definition for Omicron (eTable 4 in the Supplement), VE estimates against severe outcomes were higher and substantially more stable (eTable 5 in the Supplement).

## Discussion

In Ontario, Canada, between December 6 and 26, 2021, we found the estimated effectiveness of 2 doses of COVID-19 vaccines against symptomatic infection to be substantially lower for Omicron than for Delta; estimated VE against symptomatic Omicron infection was minimal starting at 60 days after a second dose, and there was no significant protection beyond 180 days. However, the estimated VE against symptomatic Omicron infection was 61% at 7 or more days following a third dose. Against severe outcomes, including hospitalization and death, the estimated VE shortly following a third dose was slightly lower for Omicron than for Delta.

Substantial waning of 2-dose VE against symptomatic infection was observed in England, with lower estimated VE against Omicron infection than Delta infection at each interval following 2 or 3 doses^9,11^; our results demonstrated a similar pattern, with a marked reduction in 2-dose (with at least 1 being an mRNA vaccine) and 3-dose estimated effectiveness against symptomatic Omicron infection compared with Delta infection. In data from the UK Health Security Agency, estimated VE against symptomatic Omicron infection after 2 doses of BNT162b2 or mRNA-1273 vaccine decreased to less than 20% starting at 20 weeks after a second dose.^9^ After a third dose of mRNA vaccine, estimated VE increased to approximately 60% to 75% during the first 4 weeks,^9^ consistent with our finding of 61% estimated VE 7 or more days after a third dose. Although the UK data suggest that waning VE also occurs after a third dose, insufficient time elapsed for enough third-dose recipients in Ontario to assess this in our study. In Scotland, a third dose was associated with a two-thirds reduction in the odds of symptomatic Omicron infection compared with the odds 25 or more weeks after a second dose in the early Omicron period.^13^

Although prior studies have demonstrated reduced neutralizing antibodies against Omicron compared with other variants following receipt of 2 mRNA vaccine doses^4,5,7,29,30^ (but with potent neutralization following a third dose^31,32,33^), CD8+ cytotoxic T cells are less affected by sequence variations in the Omicron variant and likely continue to provide protection against severe disease.^32,34^ Emerging clinical data have demonstrated that vaccine protection is more preserved against severe outcomes than against infection in the Omicron era. In South Africa, estimated VE against hospitalization was 93% in the pre-Omicron period and 70% in the Omicron period.^14^ In England, estimated VE against hospitalization associated with Omicron also appeared to be better maintained compared with VE against symptomatic infection with Omicron.^8,9,35^ In an analysis of the BNT162b2, mRNA-1273, and ChAdOx1 vaccines, estimated VE against Omicron-related hospitalization decreased over time after a second dose but to a lesser extent than against symptomatic infection.^9^ After a third dose, estimated VE was restored to 85% to 95% during the first 3 months.^9^ In California, estimated VE against Omicron-related hospitalization was 89% in the first 3 months following 3 doses of the BNT162b2 vaccine.^10^ In another study in the US, 3-dose VE against hospitalization when Omicron was predominant was estimated at 90%.^15^ Our 3-dose VE estimates against severe outcomes were similar to these results from England and the US. Although our 2-dose estimates against severe Omicron-related outcomes were challenging to compare given the wide 95% CIs, they suggest less waning against hospitalization than has been observed elsewhere, and this finding is consistent with surveillance data in Ontario.^36,37^

Direct comparisons with other jurisdictions are challenging^38^ owing to differences in study methods, outcome definitions (ie, identification of Omicron and Delta based on laboratory confirmation vs time-based criteria), intervals following the latest dose, vaccination policies (eg, homologous vs heterologous vaccine schedules, third-dose eligibility criteria, and product-specific policies [eg, use of mRNA vs viral vector vaccines and a preferential recommendation in Ontario for the BNT162b2 vaccine for young adults]),^39^ population age structures, public health measures implemented during the study period (eg, vaccine certificates, mask mandates^40^), testing patterns, and use of antivirals and other therapies. Furthermore, Ontario has experienced a lower cumulative incidence of reported infections and has attained higher vaccine coverage than other countries that have estimated VE against Omicron to date,^41^ and thus, it has a potentially dissimilar distribution of infection-induced vs vaccine-induced immunity.^41,42^ Despite this, the general trends across the studies are similar and suggest immune evasion, in particular in relation to infection, by Omicron.^8,9,10,13,15,16,43^

### Limitations

This study has limitations. First, we were unable to differentiate individuals who received a third vaccine dose as part of an extended primary series (ie, severely or moderately immunocompromised individuals) as well as individuals at higher risk for infection and severe infection who were eligible for a third dose earlier (eg, residents of retirement homes). Because third-dose eligibility did not expand to all adults until mid-December 2021, a proportion of third-dose recipients in this study may reflect these populations that were at higher risk; thus, our 3-dose VE estimates may be lower than what would be expected for the general population. Third-dose eligibility and timing of primary series completion may have resulted in differing characteristics of individuals by interval since last dose. Second, not all specimens were screened for SGTF owing to the rapid increase in case volumes and eligibility criteria for screening, and whole genome sequencing results identified during our study period may not have been available at the time of analysis owing to processing delays. In a sensitivity analysis for severe outcomes, we obtained higher VE estimates when we classified all cases after a certain date as Omicron. However, this less specific outcome may overestimate VE if there was differential misclassification of the variant based on vaccination status (ie, if Delta circulated at a higher level in unvaccinated individuals, VE would be overestimated). Third, changes in testing patterns, including increased use of rapid antigen tests (which were not captured in our data) and decreased PCR testing availability, may have impacted our estimates, but the direction of any resulting bias is uncertain. However, if vaccinated individuals were more likely than unvaccinated individuals to be tested, VE would be underestimated.^44^ Fourth, symptom information was not available for all laboratories submitting to Ontario’s centralized system; as such, symptomatic individuals who were tested but did not have this information recorded would have been excluded from our study.^22^ Fifth, we were unable to estimate VE for extended periods following a third dose. Sixth, there may be residual confounding that was not accounted for in our analysis. This includes an inability to control for previous undocumented infections, which may have been differential by vaccination status, and confounding due to behavioral patterns.

## Conclusions

In this test-negative case-control study, estimated VE was high against symptomatic Delta infection and severe outcomes after 2 doses of COVID-19 vaccine but was lower and more short term against symptomatic Omicron infection and better maintained against severe outcomes. A third dose was associated with improved estimated VE against symptomatic infection and with high estimated VE against severe outcomes associated with both variants. These results suggest that a third vaccine dose is important to bolster protection against both Omicron infection and severe outcomes associated with Omicron infection, although the duration of this protection is uncertain. Our work adds to a rapidly evolving body of evidence that suggests that vaccine-induced protection depends on a variety of factors—in particular, time since latest dose and circulating variant. Tools beyond the currently available vaccines, such as public health measures, antivirals or other therapies, and new formulations of COVID-19 vaccines, are likely needed to prevent Omicron infection, although protection against severe outcomes appears to be maintained with current vaccines.

## Notes

Supplement. **eFigure.** Flow Diagram **eTable 1.** Descriptive Characteristics of Participants Tested for SARS-CoV-2 With COVID-19–Relevant Symptoms During the Period December 6 to 26, 2021, Comparing Vaccinated and Unvaccinated Participants, Delta Cases and SARS-CoV-2–Negative Controls Only **eTable 2.** Parameter Estimates From Adjusted Models for Primary Analysis of Vaccine Effectiveness Against Omicron and Delta Symptomatic Infection **eTable 3.** Parameter Estimates From Adjusted Models for Primary Analysis Of Vaccine Effectiveness Against Omicron and Delta Severe Outcomes **eTable 4.** Number of Omicron and Delta Severe Outcomes (Hospitalization or Death) by Laboratory Testing Categorization During the Period December 6 to 26, 2021 **eTable 5.** Estimates of Vaccine Effectiveness Against Severe Outcomes (Hospitalization or Death) During the Period December 6 to 26, 2021, Using the Less Specific Definition of Omicron and Delta
