# Supplementary material for: Estimated Effectiveness of COVID-19 Vaccines Against Omicron or Delta Symptomatic Infection and Severe Outcomes
Source: JAMA Netw Open. 2022 Sep 22;5(9):e2232760. doi: 10.1001/jamanetworkopen.2022.32760 (PMC9500552; doi:10.1001/jamanetworkopen.2022.32760)
Supplement: Supplement. — eFigure. Flow Diagram eTable 1. Descriptive Characteristics of Participants Tested for SARS-CoV-2 With COVID-19–Relevant Symptoms During the Period December 6 to 26, 2021, Comparing Vaccinated and Unvaccinated Participants, Delta Cases and SARS-CoV-2–Negative Controls Only eTable 2. Parameter Estimates From Adjusted Models for Primary Analysis of Vaccine Effectiveness Against Omicron and Delta Symptomatic Infection eTable 3. Parameter Estimates From Adjusted Models for Primary Analysis Of Vaccine Effectiveness Against Omicron and Delta Severe Outcomes eTable 4. Number of Omicron and Delta Severe Outcomes (Hospitalization or Death) by Laboratory Testing Categorization During the Period December 6 to 26, 2021 eTable 5. Estimates of Vaccine Effectiveness Against Severe Outcomes (Hospitalization or Death) During the Period December 6 to 26, 2021, Using the Less Specific Definition of Omicron and Delta [file jamanetwopen-e2232760-s001.pdf]

Supplementary Online Content

Buchan SA, Chung H, Brown KA, et al. Estimated effectiveness of COVID-19 vaccines against Omicron or Delta symptomatic infection and severe outcomes. *JAMA Netw Open*. 2022;5(9):e2232760. doi:10.1001/jamanetworkopen.2022.32760

eFigure. Flow Diagram

eTable 1. Descriptive Characteristics of Participants Tested for SARS-CoV-2 With COVID-19–Relevant Symptoms During the Period December 6 to 26, 2021, Comparing Vaccinated and Unvaccinated Participants, Delta Cases and SARS-CoV-2–Negative Controls Only

eTable 2. Parameter Estimates From Adjusted Models for Primary Analysis of Vaccine Effectiveness Against Omicron and Delta Symptomatic Infection

eTable 3. Parameter Estimates From Adjusted Models for Primary Analysis Of Vaccine Effectiveness Against Omicron and Delta Severe Outcomes

eTable 4. Number of Omicron and Delta Severe Outcomes (Hospitalization or Death) by Laboratory Testing Categorization During the Period December 6 to 26, 2021

eTable 5. Estimates of Vaccine Effectiveness Against Severe Outcomes (Hospitalization or Death) During the Period December 6 to 26, 2021, Using the Less Specific Definition of Omicron and Delta

This supplementary material has been provided by the authors to give readers additional information about their work.

© 2022 Buchan SA et al. *JAMA Network Open*.

eFigure. Flow diagram

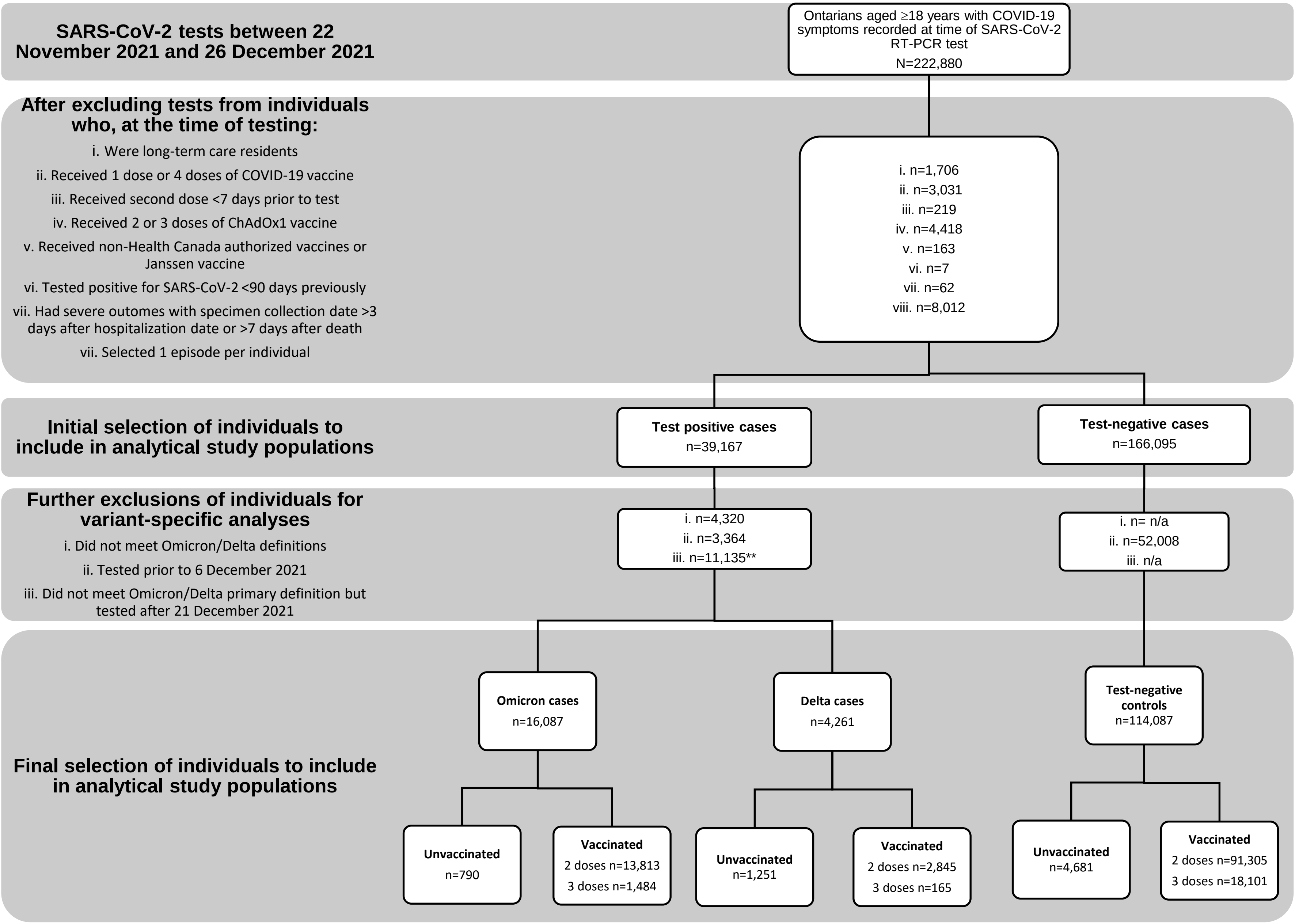

© 2022 Buchan SA et al. *JAMA Network Open*.

**Individuals were excluded from primary analysis but were included in sensitivity analysis with less specific outcome definition.

© 2022 Buchan SA et al. *JAMA Network Open*.

eTable 1. Descriptive characteristics of participants tested for SARS-CoV-2 with COVID-19–relevant symptoms during the period December 6 to 26, 2021, comparing vaccinated and unvaccinated participants, Delta cases and SARS-CoV-2–negative controls only

	**Unvaccinated, n (%)[a]**	**2 doses, n (%)[a]**	**SD[b]**	**3 doses, n (%)[a]**	**SD[b]**
**Total**	N=5,932	N=94,150		N=18,266	
Subject characteristics					
Age (years), mean (standard deviation)	38.9 ± 15.8	40.2 ± 15.3	0.09	52.6 ± 18.8	0.79
Age group (years)					
18–29	2,016 (34.0%)	27,376 (29.1%)	0.11	2,515 (13.8%)	0.49
30–39	1,637 (27.6%)	24,615 (26.1%)	0.03	3,076 (16.8%)	0.26
40–49	902 (15.2%)	16,612 (17.6%)	0.07	2,344 (12.8%)	0.07
50–59	645 (10.9%)	13,457 (14.3%)	0.10	3,272 (17.9%)	0.20
60–69	423 (7.1%)	8,188 (8.7%)	0.06	2,948 (16.1%)	0.28
70–79	199 (3.4%)	2,738 (2.9%)	0.03	2,718 (14.9%)	0.41
≥80	110 (1.9%)	1,164 (1.2%)	0.05	1,393 (7.6%)	0.27
Male sex	2,825 (47.6%)	39,011 (41.4%)	0.12	6,429 (35.2%)	0.25
Any comorbidity[c]	2,396 (40.4%)	38,250 (40.6%)	0	10,453 (57.2%)	0.34
Number of SARS-CoV-2 tests within 3 months prior to 14 Dec 2020					
0	4,847 (81.7%)	69,875 (74.2%)	0.18	12,208 (66.8%)	0.35
1	825 (13.9%)	17,984 (19.1%)	0.14	3,666 (20.1%)	0.16
≥2	260 (4.4%)	6,291 (6.7%)	0.10	2,392 (13.1%)	0.31
Receipt of 2019-2020 and/or 2020-2021 influenza vaccination	455 (7.7%)	30,775 (32.7%)	0.66	10,420 (57.0%)	1.24
Public health unit region[d]					
Central East	616 (10.4%)	7,487 (8.0%)	0.08	1,409 (7.7%)	0.09
Central West	1,123 (18.9%)	17,822 (18.9%)	0	3,110 (17.0%)	0.05
Durham	299 (5.0%)	6,573 (7.0%)	0.08	1,009 (5.5%)	0.02
Eastern	325 (5.5%)	5,707 (6.1%)	0.02	1,234 (6.8%)	0.05
North	568 (9.6%)	5,768 (6.1%)	0.13	1,495 (8.2%)	0.05
Ottawa	153 (2.6%)	4,635 (4.9%)	0.12	1,027 (5.6%)	0.15
Peel	649 (10.9%)	10,922 (11.6%)	0.02	1,547 (8.5%)	0.08
South West	1,035 (17.4%)	10,304 (10.9%)	0.19	2,363 (12.9%)	0.13
Toronto	858 (14.5%)	17,617 (18.7%)	0.11	3,799 (20.8%)	0.17
York	267 (4.5%)	6,941 (7.4%)	0.12	1,206 (6.6%)	0.09
Household income quintile[d, e]					
1 (lowest)	1,418 (23.9%)	14,532 (15.4%)	0.21	2,441 (13.4%)	0.27
2	1,295 (21.8%)	17,245 (18.3%)	0.09	3,027 (16.6%)	0.13
3	1,179 (19.9%)	18,816 (20.0%)	0	3,361 (18.4%)	0.04
4	1,071 (18.1%)	20,620 (21.9%)	0.10	4,076 (22.3%)	0.11
5 (highest)	922 (15.5%)	22,528 (23.9%)	0.21	5,270 (28.9%)	0.32
Essential workers quintile[d, f]					
1 (0%–32.5%)					
2 (32.5%–42.3%)	732 (12.3%)	21,601 (22.9%)	0.28	5,211 (28.5%)	0.41
3 (42.3%–49.8%)	1,042 (17.6%)	23,401 (24.9%)	0.18	4,573 (25.0%)	0.18
4 (50.0%–57.5%)	1,245 (21.0%)	18,893 (20.1%)	0.02	3,502 (19.2%)	0.05
5 (57.5%–100%)	1,365 (23.0%)	16,254 (17.3%)	0.14	2,829 (15.5%)	0.19
Persons per dwelling quintile[d, g]					
1 (0–2.1)	1,120 (18.9%)	17,004 (18.1%)	0.02	3,770 (20.6%)	0.04
2 (2.2–2.4)	1,261 (21.3%)	15,453 (16.4%)	0.12	3,192 (17.5%)	0.10
3 (2.5–2.6)	833 (14.0%)	12,429 (13.2%)	0.02	2,512 (13.8%)	0.01
4 (2.7–3.0)	1,424 (24.0%)	23,016 (24.4%)	0.01	4,439 (24.3%)	0.01
5 (3.1–5.7)	1,210 (20.4%)	25,632 (27.2%)	0.16	4,206 (23.0%)	0.06

© 2022 Buchan SA et al. *JAMA Network Open*.

	Unvaccinated, n (%)[a]	2 doses, n (%)[a]	SD[b]	3 doses, n (%)[a]	SD[b]
Self-identified visible minority quintile[d, h]					
1 (0.0%–2.2%)	1,225 (20.7%)	13,526 (14.4%)	0.17	2,958 (16.2%)	0.12
2 (2.2%–7.5%)	1,186 (20.0%)	16,549 (17.6%)	0.06	3,441 (18.8%)	0.03
3 (7.5%–18.7%)	1,052 (17.7%)	18,445 (19.6%)	0.05	3,921 (21.5%)	0.09
4 (18.7%–43.5%)	1,139 (19.2%)	21,562 (22.9%)	0.09	4,157 (22.8%)	0.09
5 (43.5%–100%)	1,251 (21.1%)	23,495 (25.0%)	0.09	3,645 (20.0%)	0.03
Week of test					
6 Dec 2021 to 12 Dec 2021	1,999 (33.7%)	27,997 (29.7%)	0.09	2,551 (14.0%)	0.48
13 Dec 2021 to 19 Dec 2021	2,152 (36.3%)	35,471 (37.7%)	0.03	5,297 (29.0%)	0.16
20 Dec 2021 to 26 Dec 2021	1,781 (30.0%)	30,682 (32.6%)	0.06	10,418 (57.0%)	0.57
Prior positive SARS-CoV-2 test	253 (4.3%)	3,568 (3.8%)	0.02	446 (2.4%)	0.10
COVID-19 vaccine characteristics					
Unvaccinated	5,932 (100%)	n/a		n/a	
Received 2-dose primary series only (with at least 1 mRNA vaccine)	n/a	94,150 (100%)		n/a	
Received BNT162b2 for third dose	n/a	n/a		14,916 (81.7%)	
Received mRNA-1273 for third dose	n/a	n/a		3,350 (18.3%)	
Time since second dose					
7-59 days	n/a	2,315 (2.5%)		n/a	
60-119 days	n/a	6,954 (7.4%)		n/a	
120-179 days	n/a	62,577 (66.5%)		n/a	
180-239 days	n/a	20,509 (21.8%)		n/a	
≥240 days	n/a	1,795 (1.9%)		n/a	
Time since third dose					
0-6 days	n/a	n/a		6,037 (33.1%)	
7-59 days	n/a	n/a		11,357 (62.2%)	
≥60 days	n/a	n/a		872 (4.8%)	
Interval between first and second doses					
15-34 days	n/a	12,675 (13.5%)		3,224 (17.7%)	
35-55 days	n/a	34,995 (37.2%)		3,965 (21.7%)	
≥56 days	n/a	46,480 (49.4%)		11,077 (60.6%)	
Interval between second and third doses					
≤111 days	n/a	n/a		240 (1.3%)	
112-167 days	n/a	n/a		1,643 (9.0%)	
≥168 days	n/a	n/a		16,383 (89.7%)	

[a]Proportion reported, unless stated otherwise.
[b]SD=standardized difference. Standardized differences of >0.10 are considered clinically relevant. Comparison of subjects who have received 2 doses with unvaccinated subjects, and subjects who have received 3 doses with unvaccinated subjects.
[c]Comorbidities include chronic respiratory diseases, chronic heart diseases, hypertension, diabetes, immunocompromising conditions due to underlying diseases or therapy, autoimmune diseases, chronic kidney disease, advanced liver disease, dementia/frailty and history of stroke or transient ischemic attack.
[d]The sum of counts does not equal the column total because of individuals with missing information (<1.0%) for this characteristic.
[e]Household income quintile has variable cut-off values in each city/Census area to account for cost of living. A dissemination area (DA) being in quintile 1 means it is among the lowest 20% of DAs in its city by income.
[f]Percentage of people in the area working in the following occupations: sales and service occupations; trades, transport and equipment operators and related occupations; natural resources, agriculture, and related production occupations; and occupations in manufacturing and utilities. Census counts for people are randomly rounded up or down to the nearest number divisible by 5, which causes some minor imprecision.
[g]Range of persons per dwelling.
[h]Percentage of people in the area who self-identified as a visible minority. Census counts for people are randomly rounded up or down to the nearest number divisible by 5, which causes some minor imprecision.

© 2022 Buchan SA et al. *JAMA Network Open*.

eTable 2. Parameter estimates from adjusted models for primary analysis of vaccine effectiveness against Omicron and Delta symptomatic infection

	Omicron			Delta		
**Variable**	**Estimate**	**Standard Error**	**P value**	**Estimate**	**Standard Error**	**P value**
**Intercept**	-3.6226	0.0848	<.0001	-1.7072	0.1143	<.0001
Time since vaccination						
Unvaccinated (reference)						
Dose 2: 7-59 days	-0.4408	0.0830	<.0001	-2.2211	0.1344	<.0001
Dose 2: 60-119 days	-0.1319	0.0543	0.0151	-2.1776	0.0818	<.0001
Dose 2: 120-179 days	-0.1631	0.0430	0.0001	-2.0946	0.0423	<.0001
Dose 2: 180-239 days	-0.0135	0.0457	0.7670	-1.9028	0.0547	<.0001
Dose 2: 240+ days	-0.0157	0.0869	0.8569	-1.5862	0.1254	<.0001
Dose 3: 0-6 days	-0.4495	0.0577	<.0001	-2.8520	0.1247	<.0001
Dose 3: 7+ days	-0.9424	0.0592	<.0001	-3.5120	0.1146	<.0001
Age group (years)						
18–29 (reference)						
30–39	-0.5331	0.0236	<.0001	0.1310	0.0483	0.0066
40–49	-0.3523	0.0255	<.0001	0.4621	0.0500	<.0001
50–59	-0.5821	0.0294	<.0001	0.4591	0.0540	<.0001
60–69	-1.0087	0.0434	<.0001	0.5161	0.0633	<.0001
70–79	-1.1549	0.0723	<.0001	0.9654	0.0798	<.0001
≥80	-1.0877	0.1034	<.0001	0.8224	0.1129	<.0001
Sex						
Female (reference)						
Male	0.3061	0.0179	<.0001	0.2135	0.0326	<.0001
Any comorbidity						
No (reference)						
Yes	-0.0224	0.0192	0.2425	-0.0250	0.0352	0.4780
Number of SARS-CoV-2 tests within 3 months prior to 14 Dec 2020						
0 (reference)						
1	-0.1415	0.0235	<.0001	-0.2009	0.0459	<.0001
≥2	-0.2328	0.0396	<.0001	-0.4646	0.0823	<.0001
Receipt of 2019-2020 and/or 2020-2021 influenza vaccination						
No (reference)						
Yes	-0.2830	0.0212	<.0001	-0.2533	0.0404	<.0001
Public health unit region[d]						
Central East (reference)						
Central West	0.4825	0.0431	<.0001	0.1260	0.0675	0.0619
Durham	0.2134	0.0530	<.0001	-0.3432	0.0957	0.0003
Eastern	0.3576	0.0537	<.0001	0.0688	0.0869	0.4286
North	-0.9319	0.0746	<.0001	0.0401	0.0837	0.6320
Ottawa	0.3550	0.0564	<.0001	-0.3813	0.1235	0.0020
Peel	0.5569	0.0496	<.0001	0.1460	0.0844	0.0837
South West	-0.1947	0.0510	0.0001	0.4872	0.0678	<.0001
Toronto	0.2390	0.0488	<.0001	-0.0742	0.0836	0.3751
York	0.3391	0.0524	<.0001	0.0124	0.0923	0.8932
Missing	0.1608	0.4436	0.7170	0.2255	0.6790	0.7399
Household income quintile[d, e]						
1 (lowest) (reference)						
2	0.0439	0.0351	0.2104	-0.1313	0.0562	0.0195
3	0.0864	0.0370	0.0197	-0.1167	0.0612	0.0565
4	0.1680	0.0395	<.0001	-0.1259	0.0665	0.0583

© 2022 Buchan SA et al. *JAMA Network Open*.

	Omicron			Delta		
**Variable**	**Estimate**	**Standard Error**	**P value**	**Estimate**	**Standard Error**	**P value**
5 (highest)	0.2615	0.0435	<.0001	-0.1584	0.0747	0.0340
Missing	0.1623	0.4734	0.7316	-0.5845	0.6863	0.3944
Essential workers quintile[d, f]						
1 (0%–32.5%) (reference)						
2 (32.5%–42.3%)	0.00986	0.0267	0.7124	0.1593	0.0567	0.0049
3 (42.3%–49.8%)	-0.0490	0.0312	0.1169	0.1745	0.0614	0.0045
4 (50.0%–57.5%)	-0.1047	0.0358	0.0034	0.1333	0.0667	0.0457
5 (57.5%–100%)	-0.1727	0.0418	<.0001	0.2469	0.0721	0.0006
Missing	-6.2477	56.0660	0.9113	1.6525	1.3362	0.2162
Persons per dwelling quintile[d, g]						
1 (0–2.1) (reference)						
2 (2.2–2.4)	-0.0817	0.0346	0.0182	0.00880	0.0581	0.8796
3 (2.5–2.6)	0.0110	0.0363	0.7623	0.1052	0.0642	0.1015
4 (2.7–3.0)	0.0318	0.0327	0.3304	0.1778	0.0602	0.0031
5 (3.1–5.7)	0.0297	0.0365	0.4154	0.4267	0.0676	<.0001
Missing	0.4196	0.2895	0.1473	1.0095	0.5976	0.0912
Self-identified visible minority quintile[d, h]						
1 (0.0%–2.2%) (reference)						
2 (2.2%–7.5%)	0.0693	0.0377	0.0661	0.0452	0.0536	0.3991
3 (7.5%–18.7%)	0.1801	0.0380	<.0001	0.0124	0.0582	0.8318
4 (18.7%–43.5%)	0.1513	0.0403	0.0002	-0.1672	0.0652	0.0103
5 (43.5%–100%)	0.1568	0.0459	0.0006	-0.1859	0.0781	0.0174
Missing	6.1356	56.0657	0.9129	-2.2019	1.2285	0.0731
Week of test						
6 Dec 2021 to 12 Dec 2021 (ref.)						
13 Dec 2021 to 19 Dec 2021	1.6316	0.0407	<.0001	0.0346	0.0376	0.3578
20 Dec 2021 to 26 Dec 2021	2.3534	0.0397	<.0001	-0.5167	0.0442	<.0001
Prior positive SARS-CoV-2 test						
No (reference)						
Yes	-1.7665	0.0955	<.0001	-2.8357	0.3181	<.0001

© 2022 Buchan SA et al. *JAMA Network Open*.

eTable 3. Parameter estimates from adjusted models for primary analysis of vaccine effectiveness against Omicron and Delta severe outcomes

	Omicron			Delta		
**Variable**	**Estimate**	**Standard Error**	**P value**	**Estimate**	**Standard Error**	**P value**
**Intercept**	-11.5695	1.0753	<.0001	-5.7068	0.5282	<.0001
Time since vaccination						
Unvaccinated (reference)						
Dose 2: 7-59 days	-0.8040	0.7789	0.3020	-2.8132	0.5113	<.0001
Dose 2: 60-119 days	-0.4599	0.5079	0.3652	-3.8438	0.5090	<.0001
Dose 2: 120-179 days	-1.3830	0.3462	<.0001	-4.3172	0.1927	<.0001
Dose 2: 180-239 days	-1.7125	0.3819	<.0001	-3.5588	0.2140	<.0001
Dose 2: 240+ days	-1.9924	1.0734	0.0634	-3.0673	0.5952	<.0001
Dose 3: 0-6 days	-2.3892	0.5941	<.0001	-4.3575	0.4308	<.0001
Dose 3: 7+ days	-2.9231	0.4437	<.0001	-4.5961	0.2893	<.0001
Age group (years)						
18–29 (reference)						
30–39	0.4033	0.6071	0.5065	0.8848	0.4349	0.0419
40–49	0.5590	0.6348	0.3786	2.0973	0.3984	<.0001
50–59	1.3463	0.5559	0.0154	2.8208	0.3872	<.0001
60–69	1.1019	0.6486	0.0893	3.5825	0.3836	<.0001
70–79	3.5778	0.5283	<.0001	4.2324	0.3920	<.0001
≥80	4.6432	0.5234	<.0001	4.6536	0.4047	<.0001
Sex						
Female (reference)						
Male	0.3641	0.2183	0.0953	0.5035	0.1261	<.0001
Any comorbidity						
No (reference)						
Yes	1.4566	0.3868	0.0002	0.6030	0.1595	0.0002
Number of SARS-CoV-2 tests within 3 months prior to 14 Dec 2020						
0 (reference)						
1	-0.0218	0.3326	0.9478	-0.1350	0.1984	0.4960
≥2	0.4492	0.4171	0.2815	-0.5364	0.3400	0.1146
Receipt of 2019-2020 and/or 2020-2021 influenza vaccination						
No (reference)						
Yes	-0.1780	0.2486	0.4740	-0.00616	0.1613	0.9695
Public health unit region[d]						
Central East (reference)						
Central West	0.6680	0.5685	0.2400	-0.0175	0.2466	0.9433
Durham	-0.0543	0.8000	0.9459	-0.7117	0.4417	0.1071
Eastern	0.3904	0.7202	0.5878	0.6469	0.2799	0.0208
North	-1.6170	1.1296	0.1523	-0.3383	0.2970	0.2547
Ottawa	1.1131	0.7191	0.1217	-0.2877	0.4965	0.5623
Peel	0.0426	0.6667	0.9490	-0.7776	0.3758	0.0385
South West	-0.8306	0.6875	0.2270	0.1568	0.2418	0.5168
Toronto	0.6847	0.6309	0.2777	-0.1216	0.3212	0.7051
York	0.00305	0.7723	0.9968	0.0188	0.3721	0.9596
Missing	8.0708	150.5	0.9572	10.8880	452.1	0.9808
Household income quintile[d, e]						
1 (lowest) (reference)						
2	-0.3680	0.3766	0.3286	-0.0102	0.1862	0.9562
3	0.2571	0.4055	0.5260	-0.3370	0.2267	0.1372
4	0.4584	0.4489	0.3072	-0.2797	0.2442	0.2521

© 2022 Buchan SA et al. *JAMA Network Open*.

	Omicron			Delta		
**Variable**	**Estimate**	**Standard Error**	**P value**	**Estimate**	**Standard Error**	**P value**
5 (highest)	0.4341	0.5243	0.4077	-0.7971	0.2910	0.0062
Missing	1.1156	171.4	0.9948	6.9457	999.8	0.9945
Essential workers quintile[d, f]						
1 (0%–32.5%) (reference)						
2 (32.5%–42.3%)	0.2322	0.3910	0.5525	-0.0479	0.2425	0.8433
3 (42.3%–49.8%)	0.6456	0.4109	0.1162	0.2036	0.2428	0.4018
4 (50.0%–57.5%)	0.8278	0.4478	0.0645	-0.1680	0.2631	0.5231
5 (57.5%–100%)	1.0381	0.4996	0.0377	0.0261	0.2728	0.9238
Missing	-1.6638	939.1	0.9986	15.1652	744.1	0.9837
Persons per dwelling quintile[d, g]						
1 (0–2.1) (reference)						
2 (2.2–2.4)	-0.1597	0.3820	0.6759	0.1985	0.1951	0.3090
3 (2.5–2.6)	0.0802	0.4010	0.8415	0.2449	0.2293	0.2855
4 (2.7–3.0)	-0.1603	0.3807	0.6737	0.2926	0.2233	0.1901
5 (3.1–5.7)	-0.1696	0.4476	0.7047	0.6269	0.2613	0.0164
Missing	-5.0282	70.13	0.9428	-10.6735	744.1	0.9886
Self-identified visible minority quintile[d, h]						
1 (0.0%–2.2%) (reference)						
2 (2.2%–7.5%)	0.1566	0.5067	0.7572	0.2509	0.1867	0.1790
3 (7.5%–18.7%)	0.5034	0.5074	0.3212	0.1072	0.2139	0.6164
4 (18.7%–43.5%)	1.1307	0.5212	0.0300	-0.1816	0.2497	0.4670
5 (43.5%–100%)	1.1205	0.5994	0.0616	-0.3914	0.3134	0.2116
Missing	0.5230	940.0	0.9996	-22.4921	891.7	0.9799
Week of test						
6 Dec 2021 to 12 Dec 2021 (ref.)						
13 Dec 2021 to 19 Dec 2021	1.3092	0.4268	0.0022	-0.1928	0.1427	0.1766
20 Dec 2021 to 26 Dec 2021	2.0724	0.4105	<.0001	-0.8025	0.1694	<.0001
Prior positive SARS-CoV-2 test						
No (reference)						
Yes	-0.7232	0.7321	0.3232	-1.6040	0.7243	0.0268

© 2022 Buchan SA et al. *JAMA Network Open*.

eTable 4. Number of Omicron and Delta severe outcomes (hospitalization or death) by laboratory testing categorization during the period December 6 to 26, 2021

**Classification criteria**	**6 Dec to 12 Dec 2021**	**13 Dec to 19 Dec 2021**	**20 Dec to 26 Dec 2021**
Omicron confirmed via WGS or SGTF	7	28	53
Omicron classified if collection date ≥ 21 December 2021 and no or inconclusive SGTF results	n/a	n/a	177
Delta confirmed via WGS or SGTF	121	115	61
No classification (excluded)	62	65	15

WGS: whole genome sequencing; SGTF: S-gene target failure

© 2022 Buchan SA et al. *JAMA Network Open*.

eTable 5. Estimates of vaccine effectiveness* against severe outcomes (hospitalization or death) during the period December 6 to 26, 2021, using the less specific definition of Omicron and Delta

Outcome	Doses	Vaccine products	Days since latest dose	Vaccine effectiveness against Omicron (95% CI)
Severe outcomes	First 2 doses	≥1 mRNA vaccine	7-59	89 (64, 97)
			60-119	85 (69, 92)
			120-179	92 (89, 95)
			180-239	93 (89, 95)
			≥240	93 (77, 98)
	Third dose	Any mRNA vaccine	0-6	94 (90, 96)
			≥7	98 (97, 99)
		BNT162b2	0-6	95 (91, 97)
			≥7	98 (97, 99)
		mRNA-1273	0-6	91 (81, 96)
			≥7	98 (96, 99)

*Vaccine effectiveness estimates adjusted for: age (in 10-year age bands), sex, public health unit region of residence, number of SARS-CoV-2 PCR tests during the 3 months prior to December 14, 2020, past SARS-CoV-2 infection >90 days prior to index date, comorbidities, influenza vaccination status during the 2019/2020 and/or 2020/2021 influenza seasons, and neighbourhood-level information on median household income, proportion of the working population employed as non-health essential workers, mean number of persons per dwelling, and proportion of the population who self-identify as a visible minority.

© 2022 Buchan SA et al. *JAMA Network Open*.
